# Sorption, Solubility, Bond Strength and Hardness of Denture Soft Lining Incorporated with Silver Nanoparticles

**DOI:** 10.3390/ijms14010563

**Published:** 2012-12-27

**Authors:** Grzegorz Chladek, Jacek Kasperski, Izabela Barszczewska-Rybarek, Jarosław Żmudzki

**Affiliations:** 1 Division of Materials Processing Technology, Institute of Engineering Materials and Biomaterials, Silesian University of Technology, ul. Konarskiego 18a, Gliwice 44-100, Poland; E-Mail: Jaroslaw.Zmudzki@polsl.pl; 2 Department of Prosthetic Dentistry, Medical University of Silesia, pl. Akademicki 17, Bytom 41-902, Poland; E-Mail: kroczek91@interia.pl; 3 Department of Physical Chemistry and Technology of Polymers, Silesian University of Technology, ul. M. Strzody 9, Gliwice 44-100, Poland; E-Mail: Izabela.Barszczewska-Rybarek@polsl.pl

**Keywords:** antimicrobial polymers, silver nanoparticles, soft lining, bond strength, sorption, solubility, hardness

## Abstract

The colonization of denture soft lining material by oral fungi can result in infections and stomatitis of oral tissues. In this study, 0 ppm to 200 ppm of silver nanoparticles was incorporated as an antimicrobial agent into composites to reduce the microbial colonization of lining materials. The effect of silver nanoparticle incorporation into a soft lining material on the sorption, solubility, hardness (on the Shore A scale) and tensile bond strength of the composites was investigated. The data were statistically analyzed using two-way ANOVA and Newman-Keuls *post hoc* tests or the chi-square Pearson test at the *p* < 0.05 level. An increase in the nanosilver concentration resulted in a decrease in hardness, an increase in sorption and solubility, a decrease in bond strength and a change in the failure type of the samples. The best combination of bond strength, sorption, solubility and hardness with antifungal efficacy was achieved for silver nanoparticle concentrations ranging from 20 ppm to 40 ppm. These composites did not show properties worse than those of the material without silver nanoparticles and exhibited enhanced *in vitro* antifungal efficiency.

## 1. Introduction

Permanent soft denture lining materials bonded to dentures are usually used to reduce the forces transmitted to supporting tissues. They are recommended for patients suffering from a sharp alveolar ridge or a thin atrophic mucosa, in which the mucosa shows low tolerance to the load applied by dentures and for relining in implantology [1].

One of the basic problems with using soft denture linings is the colonization of such materials by pathological microorganisms [1–3], which is fostered by the high humidity and elevated temperature found under dentures and by the material structure [4]. Fungi such as *Candida albicans* first adhere to a lining surface and then penetrate inside the material [1], which can lead to further infection of the oral mucosa and problems associated with stomatitis. Meanwhile, silver nanoparticles [5–7] and silver-zeolite [8] show *in vitro* antimicrobial effects as additives in tissue conditioners and denture base acrylic resins. Therefore, a method of incorporating silver nanoparticles into silicone soft lining materials to enhance antimicrobial efficacy was developed; the fungicidal activity of the obtained composites was confirmed in a previous study [9].

However, soft lining materials exposed to the continual influence of a humid environment can lose their soluble components [10] and absorb water [11]. The changes induced by the sorption, solubility, dehydration and oxidization of materials in a humid environment can result in the deterioration of mechanical and functional properties such as hardness and bond strength [12–14].

The purpose of this study was to evaluate the impact of silver nanoparticle incorporation into composites on the materials’ sorption, solubility, hardness and tensile bond strength. The hypothesis was that, after aging in distilled water, the hardness, water sorption, solubility, and tensile bond strength of the composites depend on the concentration of silver nanoparticles.

## 2. Results and Discussion

### 2.1. Results

Introducing silver nanoparticles into Ufi Gel SC (UG) soft liner material resulted in the prolongation of the cross-linking time of the samples. The cross-linking time was observed to increase with the silver nanoparticle concentration. The cross-linking time for a composite with 40 ppm of silver nanoparticles was approximately 15 min longer than that of UG, but for a material with 80 ppm of silver nanoparticles, the cross-linking time was approximately 40 min longer. For samples featuring a silver nanoparticle concentration of 200 ppm, the cross-linking time was over 2 h.

The nanosilver concentration had a significant influence on the mean hardness values of the composites (*p* < 0001). The hardness was observed to increase with the silver nanoparticle concentration. The aging time had no effect on hardness (*p* = 0.7547). The mean hardness values are listed in Table 1.

The nanosilver concentration and aging time had a significant influence on the mean sorption values of the composites (*p* < 0.0001). The sorption increased with the aging time and the silver nanoparticle concentration, but the impact of the aging time was stronger than that of the silver nanoparticle concentration. The mean sorption values are listed in Table 2. *Post hoc* tests showed no statistically significant differences between the mean sorption values of UG and the nanocomposites’ nanosilver concentrations from 10 ppm to 40 ppm, regardless of the aging time used. Increasing the silver nanoparticle concentration above 80 ppm caused an increase in sorption (*p* < 0.05). There were no significant differences (*p* > 0.05) in the sorption between 7 days and 28 days of aging up to a concentration of 40 ppm, but above 80 ppm, the sorption after 28 days was greater (*p* < 0.05) than that after 7 days.

The silver nanoparticle concentration and aging time had a significant influence on the solubility of the composites (*p* < 0.0001 and *p* = 0.0039, respectively). The solubility was observed to increase with the aging time and the silver nanoparticle concentration, and the effect of the aging time was weaker than that of the silver nanoparticle concentration. The mean solubility values are listed in Table 2. Statistically significant differences between the mean values of solubility for the samples composed of UG and composites with a silver nanoparticle concentration ranging from 10 ppm to 120 ppm and from 10 ppm to 80 ppm after 7 days and 28 days of aging, respectively, were not observed (*p* > 0.05). Only the solubility at the highest nanosilver content after 28 days was statistically greater (*p* <0 .05) than that after 7 days.

The nanosilver concentration and aging time had a significant influence on the mean bond strength values of the composites (*p* < 0.0001), but the effect of the aging time was approximately 11 times lower than that of the nanosilver concentration. The mean bond strength values are listed in Table 3. For individual aging times, the mean bond strength values for UG and the composites with nanosilver concentrations of 10, 20 and 40 ppm did not differ statistically (*p* > 0.05). Above a concentration of 80 ppm, a considerable decrease in the mean bond strength values was observed. The effect of the aging time on the mean bond strength (increase of the values) was significant (*p* < 0.05) in the case of UG and the composites with nanosilver concentrations of 10 ppm to 20 ppm, and differences were noted only between samples stored for 24 h and 7 days.

**Figure 1:**
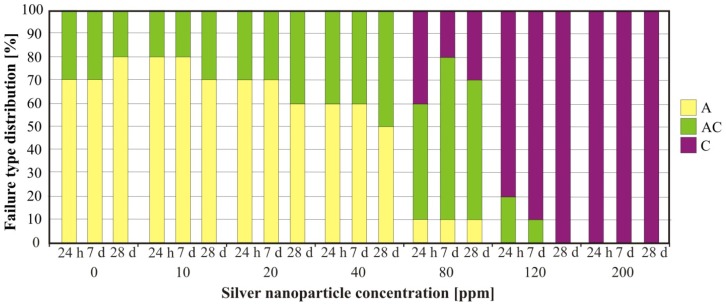
Impact of the silver nanoparticle concentration on failure type after 24 h, 7 days and 28 days of aging. A—Adhesive failure; AC—Mixed failure; C—Cohesive failure.

The nanosilver concentration had a significant influence on the sample failure type (*p* < 0.0001) (Figure 1). For UG and composites with silver nanoparticle concentrations up to 40 ppm, adhesive failure was the dominant failure type observed, whereas at higher concentrations, the failure mechanism varied from mixed (80 ppm) to cohesive (200 ppm). The aging time had no effect on the failure type (*p* > 0.05).

### 2.2. Discussion

The best combination of bond strength, sorption, solubility and hardness with antifungal efficacy was achieved at silver nanoparticle concentrations ranging from 20 ppm to 40 ppm. These composites exhibited properties that were no worse than those of UG without silver nanoparticles and also demonstrated enhanced antifungal efficiency [9]. Additionally, composites with such low silver nanoparticle concentrations should not generate cytotoxicity [15], but this assumption should be confirmed in further investigations before potential clinical investigations are carried out.

All of the obtained materials were cross-linked, and the cross-linking time increased with the silver nanoparticle concentration. The prolonging of the cross-linking time was particularly noticeable starting at a silver nanoparticle concentration of 80 ppm. A decrease in the rate of polymerization and the conversion value with the silver nanoparticle concentration in the investigated polymers was also reported in [16–18]. Soriano-Corral *et al.* [16] reported a decrease in the rate of polymerization related to the physical interaction between the silver nanoparticles and free radicals present in the reaction medium. Fan *et al.* [5] assumed that the cross-linking problems associated with dental resin, which occur with increasing silver nanoparticle concentration, can be caused by the agglomeration of the silver nanoparticles. Based on previously reported SEM examinations of studied materials [9], a significant growth in the number and size of aggregates was observed starting at a silver nanoparticle concentration of 80 ppm. This implies that there is a connection between the formation of aggregates and the considerable prolonging of the cross-linking time. The nanoparticles and aggregates may physically interact with the reactive polymer groups and catalyst particles, but this assumption requires confirmation.

The mean hardness value obtained for UG is similar to that reported by Mancuso *et al.* [13]. For the obtained composites, the hardness decreased with the increasing concentration of silver nanoparticles. However, for concentrations from 10 ppm to 40 ppm, the hardness values were greater than 25 Shore A; thus, these materials conform to the requirements of the ISO standard for soft lining materials [19], but composites with concentrations ranging from 80 ppm to 200 ppm show the hardness required for extra-soft lining materials. Changes in hardness during aging in distilled water were not observed. These results are in accordance with those of other studies that show that silicone-based materials, in contrast to acrylic-based soft linings, generally present no changes in hardness after soaking in water [20]. This can be explained by the differences in chemical composition between silicone-based and acrylic-based materials. Acrylic-based soft linings contain plasticizers that affect the initial softness of the materials [13]; thus, the loss of plasticizers causes hardening [21]. However, no plasticizer is needed to induce a softening effect in silicone-based materials because their softness is modulated by the concentration of cross-linking agent in the base rubber material [22].

Due to the problems associated with evaluating soft samples with calipers, we were forced to alter our calculations for solubility and sorption, which differed from those prescribed by ISO standards. It was noted that, during the measurements, the dimensions (particularly the diameters) of the samples were unintentionally deformed; thus, it was concluded that the results would not be accurate. Such problems do not occur when measuring sample mass; therefore, the percentage changes in mass were determined. This method of calculating the sorption and solubility has been reported in the literature [13] but does not allow for a comparison of the results with those of a standard. The mean sorption and solubility values obtained for UG and composites with silver concentrations of up to 200 ppm were smaller than those reported by Mancuso *et al.* [13]; however, their experiments were not performed after soaking in distilled water but after 2000 thermal cycles. Nevertheless, the sorption and solubility of the composites were comparable to those reported for other silicone-based materials, including the similar Ufi Gel P, after 1 week and 4 weeks of aging in distilled water [23]. Moreover, the mean sorption and solubility values of composites containing a wide range of silver nanoparticle concentrations (up to 120 ppm) were comparable to those obtained for UG. It should be emphasized that silicone-based materials generally exhibit much lower sorption and solubility than acrylic-based lining materials because they do not contain components such as plasticizers that are rinsed out by water and consequently allow water absorption [16–18,20,21,24–26].

The bond quality of lining materials with denture base materials is usually determined by three commonly accepted methods: peel, shear and tensile tests [25,26]. However, these laboratory tests do not fully reflect the clinical bond strength of soft lining materials because they only allow for the analysis of one type of material load, whereas materials in the oral cavity are exposed to various loads due to forces that act over long periods [24]. Still, these methods are especially useful for comparing the bond strength and failure type of certain lining materials. The tensile test is preferred when examining strength [26] and is standardized by the ISO [19]. Nevertheless, there may be some difficulties in interpretation, mainly due to the failure type of a given set of samples. Lining materials exhibit a relatively low tensile strength that is quite frequently lower than the bond strength. This results in cohesive or adhesive-cohesive failure in samples. In fact, the strength of a lining material or the combination of a material’s strength and bond strength is often measured instead of the bond strength alone. In such cases, the bond strength is noted to be higher than the tensile strength of the material tested.

The mean bond strength obtained for UG is similar to the values reported by Mutluay *et al.* [26] and Lassila *et al.* [27]. A significant deterioration in the mean bond strength and changes in the failure type from adhesive to mixed to cohesive were observed starting at silver nanoparticle concentrations of 80 ppm. This indicates that the tensile strength of those materials was greatly reduced. Composites containing silver nanoparticle concentrations of up to 40 ppm conform to the ISO standard requirements for soft lining materials (the bond strength after 24 h of soaking in distilled water was higher than 1 MPa in at least 8 out of each 10 samples). Materials with a silver nanoparticle concentration of 80 ppm were classified as extra soft, but materials with higher silver nanoparticle concentrations did not fulfill the ISO standard requirements.

The mean bond strength of UG and composites with silver nanoparticle concentrations of up to 40 ppm did not differ statistically. For those materials, an increase in the mean bond strength was observed after seven days of aging. Yanikogtlu *et al.* [28] also noted that, after the first seven days of aging in distilled water, artificial saliva, coffee and tea, the bond strength of Ufi Gel P material increased. Mutluay *et al.* [26] reported that silicone materials bonded with an acrylic base material previously stored in distilled water exhibited a higher bond strength than that of a dry acrylic substrate. They concluded that this phenomenon could be caused by the interfacial reactions that take place when wet denture base resin samples were used. These reactions can lead to interpenetration and cross-linking, which result in the formation of a more organized structure [27]. This may explain not only the higher bond strength obtained due to the application of wet denture base materials but also the increase in bond strength observed after seven days of aging in distilled water. The decrease in the mean bond strength values noted after 28 days was not statistically significant. The bond strength of the similar Ufi Gel P soft liner reported by Aydin *et al.* [14] and Yanikoglu *et al.* [28] after 30 days of aging in water decreased; however, Yanikoglu *et al.* [28] reported a much greater decrease in bond strength than Aydin *et al.* [14]. These differences may be due to material type, the methods of sample preparation or test parameters such as the cross-head speed of the testing machine used throughout the course of examinations.

The negative consequences of introducing nanoparticles into the composites considered in this study on the hardness, sorption, solubility and bond strength of the materials were associated with the observed prolonging of the cross-linking time. If composites with silver nanoparticle concentrations of up to 40 ppm presented properties similar to those of UG, the composites with higher concentrations of nanoparticles were even more weakly cross-linked, which adversely affected the other properties investigated. Additionally, a prolonged cross-linking time may create difficulties during a direct relining in the mouth. In any event, the results obtained in this study should be considered together with those previously reported on antifungal activity [9]. In an environment containing UG specimens, an increase of 23.4% in the CFU/mL value of Candida albicans was observed (in comparison to a positive control). This result indicates that soft liners can support fungal growth and corresponds well with the results reported by Pavan *et al.* [2]. The antifungal efficiencies (AFEs) of composites (relative to a positive control) ranged from 16.3% for a composite with 10 ppm of nanosilver to 52.2% obtained for a composite with 200 ppm of nanosilver (thus, the reduction in the presence of fungi related to UG ranged from 39.7% to 75.6%). The most effective treatment was an increase in the silver nanoparticle concentration to 40 ppm, for which the AFE value was 31.5%. Further increasing the nanosilver concentration from 40 ppm to 200 ppm was less effective: a quintuple increase in concentration increased the antifungal efficacy by only an additional 28%. The reduced effectiveness of increasing doses of silver introduced into the composites is related to the previously reported increase in the number and size of aggregates: scanning electron microscopy measurements indicated the presence of individual particles and nanoparticle aggregates in all composites; however, above a concentration of 80 ppm, the size of the aggregates that formed mostly ranged between 100 nm and 300 nm, with the size some large aggregates exceeding 1 μm [9]. The aggregation of nanosilver reduces the effective surface area of the nanoparticles and silver ion emission, which reduces the particles’ antimicrobial effect [15]. Additionally, it should be taken into account that the results of antifungal *in vitro* tests are not confirmation of clinical relevance but, together with hardness, sorption, solubility and bond strength tests, may be a good starting point for cytotoxicity and *in vivo* investigations.

## 3. Experimental Section

The composites were prepared by the previously described method [9]. The silicone soft liner UG (VOCO: Cuxhaven, Germany) and a 30 ppm (*w*/*w*) silver nanoparticle colloid in n-hexane (Amepox Ltd.: ŁódŸ, Poland) were used to prepare the composites. The average nanoparticle size of the colloid, as confirmed by dynamic light scattering (DLS), was 22.8 nm [9].

Silver nanoparticles were introduced separately into both components of the UG material: the UG base (mixture of polyalkylsiloxanes, fumed silica and pigments) and UG catalyst (mixture of polyalkylsiloxanes and catalyst). The UG components were dissolved in hexane by stirring with a magnetic stirrer at room temperature for 2 h, achieving a concentration of 7% (*w*/*w*). The silver nanoparticle colloid mass required to produce a component with a particular concentration was calculated according to the following equation:

(1) $$m_{AgH}=\frac{c_{Agm}\times m_{m}\times 10^{6}}{c_{AgH}\times (10^{6}-c_{Agm})}$$

where *m*_AgH_ is the silver nanoparticle colloid mass (g), *c*_Agm_ is the silver nanoparticle concentration in a given composite component (ppm), *m*_m_ is the UG component mass (g) and *c*_AgH_ is the silver nanoparticle concentration in the hexane colloid.

The colloid mass calculated according to Equation 1 was added to the solution of a modified component, and the mixture was stirred with a magnetic stirrer for 15 min.

Next, the hexane was evaporated from the mixtures using a two-step procedure. First, it was preliminarily evaporated under a reduced pressure of 100 mbar in a rotary evaporator (IKA RV-10 rotary evaporator equipped with Vacuubrand DVR 2 vacuum meter); then, the condensed composition was poured into a Petri dish and warmed in a dryer at 50 °C for 24 h.

According to this procedure, both the UG base and the UG catalyst were modified to obtain the following silver nanoparticle concentrations: 10, 20, 40, 80, 120 and 200 ppm. During the cross-linking of the samples, components with the same silver nanoparticle concentration were mixed together in a mass ratio of 1:1; thus, six different composites were fabricated and examined. Before testing the samples for hardness, sorption, solubility and bond strength, a simple test was carried out to define the changes in the cross-linking time of the composites. Five samples of each material (40 mm in diameter and 6 mm in thickness) were tested. Cross-linking was carried out at 45 °C, the temperature recommended by the manufacturer. During the test, measurements of sample hardness on the Shore A scale were repeated in 5 min intervals until the same value was registered four times in a row (no registered increase in hardness for 20 min). The period spanning from the start of the experiment to the final hardness measurements was accepted as the cross-linking time.

The hardness after 5 s of loading was measured using a method presented in the ISO standard [19], but in this study, measurements were taken after three storage times. An HDA 100-1 Shore A Digital Durometer (Zwick GmbH & Com: Ulm, Germany) was used to measure the hardness. Material components were mixed manually, and samples measuring 40 mm in diameter and 6 mm in thickness were created in a steel mold. Three samples were created for every material (*n* = 21). The hardness was measured after 24 h, 7 days and 28 days of aging in distilled water at 37 ± 1 °C. The hardness of every sample after each aging time was measured at five measurement points, which were at least 5 mm from the edge of each sample and spaced at least 2 mm away from each other. When the measurement was completed, the sample was immediately reimmersed in water.

The sorption and solubility of the obtained composites were determined using a method based on the ISO standard [19], with some modifications made with respect to the aging time and the method of calculation. Material components were mixed manually, and test samples measuring 50 mm in diameter and 0.5 mm in thickness were created in stainless steel molds. Cross-linked samples were removed from the mold using tweezers, and their quality was examined to determine whether the surfaces were flat or contained any bubbles. Ten samples of each material were created (*n* = 70). The samples were weighed on an AS 110/C/2 analytic scale (Radwag: Radom, Poland) with a measurement accuracy of 0.1 mg and were placed inside desiccators containing freshly dried silica gel. The desiccators were placed in a dryer at 37 ± 1 °C, and the samples were weighed every 24 h. These measurement cycles were repeated until the daily changes in mass were no higher than 0.2 mg. Stable values were registered as m_1_ “conditioned mass”, and the samples were placed in a chamber filled with distilled water (POCH: Gliwice, Poland) at 37 ± 1 °C. Two aging times were used: 7 days and 28 days. After aging, the samples were removed from water, and all visible moisture was removed using filter paper; the samples were air-dried for approximately 15 s and then weighed. The registered mass was denoted m_2_. The samples were placed in desiccators with freshly dried silica gel and dried until they reached a stable mass, denoted as m_3_. The sorption and solubility of each sample were calculated using the following equations [13]:

(2) $$w_{sp}=\frac{m_{2}-m_{3}}{m_{1}}\times 100\%$$

(3) $$w_{s1}=\frac{m_{1}-m_{3}}{m_{1}}\times 100\%$$

where *w*_sp_ is sorption, *w*_sl_ is solubility, *m*_l_ is the initial mass of dried samples, *m*_2_ is the mass after aging, and *m*_3_ is the mass after the second drying step.

The tensile bond strength between the UG liner or composites and the denture base resin was measured by a slightly modified version of the method presented in the ISO standard [19]. Heat-cured PMMA resin Vertex Rapid Simplified (Vertex-Dental B.V.: Zeist, The Netherlands) plates (100 mm × 100 mm) were prepared. The plates were preliminarily ground on abrasive papers (Struers A/S: Copenhagen, Denmark) in the grit size sequence 120, 220 and 320 to eliminate any possible unevenness and to standardize the plates’ thickness. Following this grinding process, the thickness of the plates was 3.1 ± 0.2 mm. The plates were cut into square pieces measuring 25 mm on a side. The obtained PMMA samples were thoroughly rinsed, and their working surfaces were wet-ground by 500-grit abrasive paper to remove the scratches made by the previous grinding procedure. The prepared samples were placed in distilled water and stored at 37 ± 1 °C for 28 days ± 5 h. After aging, they were taken out in pairs from the bath; the surface of the samples was dried with filter paper, and the bonding agent Ufi Gel SC Adhesive (VOCO: Cuxhaven, Germany) was applied with a brush. The first plate was placed on the compression table mounted to the testing machine. Next, a polyethylene ring with an internal diameter of 11 mm and a thickness of 3 mm was placed in the middle of the plate. The test material was manually mixed and injected into the ring; then, a second acrylic plate was placed over the material, and the material was compressed with a force of 30 N. When the tested material was cross-linked, a handle was fixed by an auto-polymer to the sample. Thirty samples were made from each material (*n* = 210). The samples were aged in distilled water at 37 ± 1 °C for 24 ± 1 h, 7 days ± 1 h and 28 days ± 2 h. As soon as the samples were taken out of the bath, they were immediately mounted in the jaws of the testing machine (Zwick GmbH & Com: Ulm, Germany), which was equipped with prepared handles, and tensile testing was performed at a cross-head speed of 10 mm/min. The bond strength σ_B_ (MPa) was calculated according to the following equation:

(4) $$\sigma_{\text{B}}=\frac{F_{\text{max}}}{A}$$

where *F*_max_ is the maximum force (N) and *A* is the initial surface area of the bond between the silicone and resin (mm^2^).

**Figure 2:**
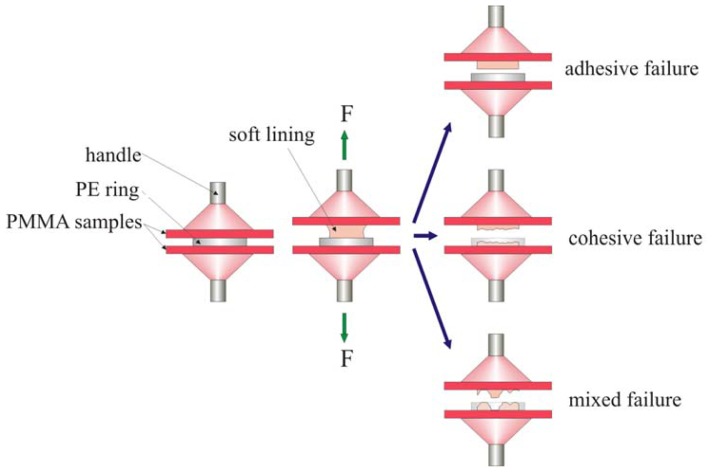
Diagrammatic presentation of the test procedure for determination of tensile bond strength and failure type.

Additionally, the failure type was defined visually for each sample [26] as being adhesive (interfacial), cohesive (complete bulk) or mixed adhesive and cohesive (Figure 2).

The results of the sorption, solubility, tensile bond strength and hardness tests were statistically evaluated using the two-way analysis of variance (ANOVA) (silver nanoparticle concentration and storage time). The Newman-Keuls significant difference *post hoc* test was used to determine the differences between mean values. Statistical significance was defined at *p* < 0.05. The impact of the silver nanoparticle concentration and the sample aging time in distilled water on the type of failure observed was defined by the chi-square (χ^2^) Pearson test. Statistical significance was defined at *p* < 0.05.

## 4. Conclusions

The hypothesis that the hardness, absorption, solubility and tensile bond strength of composites after artificial aging are dependent on silver nanoparticle concentration was confirmed. Composites with silver nanoparticle concentrations of up to 40 ppm presented properties that were no worse than those of the UG liner material. Starting at a concentration of 80 ppm, the hardness and tensile bond strength of the composites were greatly reduced, while the absorption and solubility increased due to problems associated with the cross-linking of the composites.

## Figures and Tables

**Table 1:** Mean hardness values in Shore A units and standard deviations.*

Silver nanoparticle concentration, ppm	Hardness, Shore A units		

	24 h	7 days	28 days
**0**	31.2 (0.6) ^A,a^	31.3 (0.5) ^A,a^	31.1 (0.5) ^A,a^
**10**	28.9 (0.5) ^B,b^	28.8 (0.7) ^B,b^	28.9 (0.8) ^B,b^
**20**	28.2 (0.6) ^C,bd^	28.2 (0.6) ^C,bd^	28.1 (0.7) ^C,d^
**40**	27.5 (0.7) ^D,d^	27.5 (0.7) ^D,d^	27.6 (0.7) ^D,d^
**80**	25.9 (0.4) ^E,e^	26.3 (0.5) ^E,e^	26.2 (0.8) ^E,e^
**120**	22.8 (0.4) ^F,f^	22.9 (0.5) ^F,f^	22.8 (0.5) ^F,f^
**200**	21.4 (1.0) ^G,g^	21.3 (1.1) ^G,g^	21.3 (0.8) ^G,g^

Notes:

* Groups with the same uppercase superscript letters; (A–G) for each row and lowercase superscript letters; (a–g) for each column are not significantly different at the *p* < 0.05 level.

**Table 2:** Results of sorption and solubility investigations (mean values with standard deviations).*

Silver nanoparticle concentration, ppm	Sorption, %		Solubility, %	

	7 days	28 days	7 days	28 days
**0**	0.27 (0.05) ^A,a^	0.37 (0.06) ^A,a^	0.09 (0.01) ^A,a^	0.10 (0.02) ^A,a^
**10**	0.32 (0.06) ^A,ab^	0.41 (0.06) ^A,ab^	0.09 (0.02) ^A,a^	0.11 (0.02) ^A,a^
**20**	0.41 (0.06) ^A,ab^	0.46 (0.08) ^A,ab^	0.11 (0.03) ^A,a^	0.10 (0.03) ^A,a^
**40**	0.37 (0.06) ^A,ab^	0.51 (0.09) ^A,ab^	0.09 (0.02) ^A,a^	0.09 (0.02) ^A,a^
**80**	0.38 (0.08) ^A,ab^	0.59 (0.09) ^B,b^	0.11 (0.02) ^A,a^	0.14 (0.02) ^A,ab^
**120**	0.51 (0.09) ^A,b^	0.79 (0.14) ^B,c^	0.15 (0.03) ^A,a^	0.19 (0.04) ^A,b^
**200**	0.72 (0.12) ^A,c^	1.24 (0.18) ^B,d^	0.22 (0.05) ^A,b^	0.30 (0.05) ^B,c^

Notes:

* Groups with the same uppercase superscript letters; (A–B) for each row and lowercase superscript letters; (a–d) for each column are not significantly different at the *p* < 0.05 level.

**Table 3:** Bond strength values (mean and standard deviations).*

Silver nanoparticle concentration, ppm	Bond strength, MPa		

	24 h	7 days	28 days
**0**	1.18 (0.17) ^A,a^	1.53 (0.17) ^B,a^	1.48 (0.29) ^B,a^
**10**	1.29 (0.24) ^A,a^	1.62 (0.23) ^B,a^	1.51 (0.31) ^AB,a^
**20**	1.28 (0.16) ^A,a^	1.61 (0.30) ^B,a^	1.55 (0.26) ^AB,a^
**40**	1.33 (0.22) ^A,a^	1.59 (0.36) ^A,a^	1.58 (0.37) ^A,a^
**80**	0.91 (0.15) ^A,b^	0.96 (0.14) ^A,b^	0.93 (0.11) ^A,b^
**120**	0.51 (0.06) ^A,c^	0.54 (0.02) ^A,c^	0.52 (0.02) ^A,c^
**200**	0.22 (0.03) ^A,d^	0.25 (0.02) ^A,d^	0.21 (0.02) ^A,d^

* Groups with the same uppercase superscript letters; (A–B) for each row and lowercase superscript letters; (a–d) for each column are not significantly different at the *p* > 0.05 level.
